# Expression of Concern: A novel MCGDM technique based on correlation coefficients under probabilistic hesitant fuzzy environment and its application in clinical comprehensive evaluation of orphan drugs

**DOI:** 10.1371/journal.pone.0359700

**Published:** 2026-10-12

**Authors:** The PLOS One Editors

The *PLOS One* Editors issue this Expression of Concern to inform readers that the articles cited as References 29, 86, and 88 were retracted after [1] was published.

Readers are advised to interpret the article [1] with caution.
