# Supplementary material for: A novel MCGDM technique based on correlation coefficients under probabilistic hesitant fuzzy environment and its application in clinical comprehensive evaluation of orphan drugs
Source: PLoS One. 2024 May 6;19(5):e0303042. doi: 10.1371/journal.pone.0303042 (PMC11073718; doi:10.1371/journal.pone.0303042)
Supplement: S3 Table — (DOC) [file pone.0303042.s003.doc]

**S3 Table. The individual decision matrix of D3**

|  | *A_1_* | *A_2_* | *A_3_* | *A_4_* | *A_5_* |
| --- | --- | --- | --- | --- | --- |
| *C_1_* | HVH | ML | H | MH | H |
| *C_2_* | ML | H | VH | L | MH |
| *C_3_* | H | H | M | L | H |
| *C_4_* | H | M | VLL | MH | M |
