# Supplementary material for: A novel MCGDM technique based on correlation coefficients under probabilistic hesitant fuzzy environment and its application in clinical comprehensive evaluation of orphan drugs
Source: PLoS One. 2024 May 6;19(5):e0303042. doi: 10.1371/journal.pone.0303042 (PMC11073718; doi:10.1371/journal.pone.0303042)
Supplement: S5 Table — (DOC) [file pone.0303042.s005.doc]

**S5 Table. The PHF group decision matrix.**

|  | *C_1_* | *C_2_* | *C_3_* | *C_4_* |
| --- | --- | --- | --- | --- |
| *A_1_* | 0.89|0.5,1|0.5 | 0.6|0.25,0.75|0.75 | 0.75|0.25,0.88|0.75 | 0.78|0.5,  1|0.5 |
| *A_2_* | 0.22|0.25,  0.33|0.25,0.44|0.5 | 0.33|0.25,0.43|0.25,  0.5|0.25,0.6|0.25 | 0.88|1 | 0.56|0.25,  0.67|0.25,0.44|0.5 |
| *A_3_* | 0.67|0.25,0.78|0.75 | 0.33|0.5,0.43|0.5 | 0.63|1 | 0.11|0.5,0.22|0.5 |
| *A_4_* | 0.67|0.5,0.78|0.5 | 0.75|0.5,1|0.5 | 0.38|0.5,0.5|0.5 | 0.78|0.25,0.67|0.75 |
| *A_5_* | 0.78|1 | 0.43|0.5,0.5|0.5 | 0.75|0.25,  1|0.25,0.88|0.5 | 0.44|0.5,0.56|0.5 |
