# Supplementary material for: A novel MCGDM technique based on correlation coefficients under probabilistic hesitant fuzzy environment and its application in clinical comprehensive evaluation of orphan drugs
Source: PLoS One. 2024 May 6;19(5):e0303042. doi: 10.1371/journal.pone.0303042 (PMC11073718; doi:10.1371/journal.pone.0303042)
Supplement: S8 Table — (DOC) [file pone.0303042.s008.doc]

**S8 Table. The decision matrix.**

|  | *C_1_* | *C_2_* | *C_3_* | *C_4_* | *C_5_* |
| --- | --- | --- | --- | --- | --- |
| *A_1_* | 0.7|0.25,  0.5|0.75 | 0.8|0.1,  0.6|0.25,  0.7|0.65 | 0.3|0.15,  0.6|0.25,  0.4|0.6 | 0.3|0.125,  0.4|0.125,  0.6|0.25,  0.7|0.5 | 0.3|0.25,  0.4|0.25,  0.5|0.25,  0.8|0.25 |
| *A_2_* | 0.2|0.1,  0.4|0.25,  0.3|0.65 | 0.4|0.5,  0.5|0.5 | 0.6|0.125,  0.4|0.25,  0.5|0.375,  0.8|0.25 | 0.4|0.25,  0.6|0.375,  0.7|0.375 | 0.4|0.25,  0.6|0.75 |
| *A_3_* | 0.4|0.25,  0.7|0.3,  0.6|0.45 | 0.4|0.25,  0.6|0.25,  0.5|0.5 | 0.2|0.25,  0.3|0.25,  0.5|0.25,  0.6|0.25 | 0.1|0.25,  0.5|0.25,  0.3|0.5 | 0.6|0.125,  0.4|0.5,  0.7|0.375 |
| *A_4_* | 0.2|0.25,  0.3|0.25,  0.4|0.25,  0.6|0.25 | 0.4|0.25,  0.3|0.5,  0.6|0.25 | 0.3|0.25,  0.4|0.25,  0.5|0.5 | 0.4|0.175,  0.6|0.25,  0.5|0.575 | 0.2|0.25,  0.4|0.125,  0.5|0.375,  0.7|0.25 |
