# Supplementary material for: A novel MCGDM technique based on correlation coefficients under probabilistic hesitant fuzzy environment and its application in clinical comprehensive evaluation of orphan drugs
Source: PLoS One. 2024 May 6;19(5):e0303042. doi: 10.1371/journal.pone.0303042 (PMC11073718; doi:10.1371/journal.pone.0303042)
Supplement: S9 Table — (DOC) [file pone.0303042.s009.doc]

**S9 Table. The decision matrix.**

|  | *C_1_* | *C_2_* | *C_3_* |
| --- | --- | --- | --- |
| *A_1_* | 0.76|0.1,0.55|0.15,  0.65|0.25,0.8|0.5 | 0.2|0.05,0.4|0.125,  0.3|0.325,0.65|0.25,  0.75|0.25 | 0.94|0.1,0.8|0.15,  0.75|0.25,0.55|0.5 |
| *A_2_* | 0.4|0.25,0.58|0.25,  0.69|0.25,0.95|0.25 | 0.6|0.075,0.8|0.075,  0.35|0.25,0.65|0.25,  0.7|0.35 | 0.25|0.25,0.45|0.375,  0.65|0.375 |
| *A_3_* | 0.3|0.1,0.68|0.25，  0.5|0.35,0.6|0.3 | 0.55|0.125,0.66|0.125,  0.45|0.25,0.56|0.25,  0.85|0.25 | 0.45|0.25,0.55|0.25,  0.68|0.25,0.75|0.25 |
| *A_4_* | 0.15|0.1,0.37|0.15,  0.4|0.25,0.6|0.25,  0.73|0.25 | 0.62|0.1,0.55|0.25,  0.66|0.25,0.48|0.4 | 0.5|0.125,0.7|0.125,  0.38|0.25,0.75|0.25,  0.85|0.25 |
