# Supplementary material for: A novel MCGDM technique based on correlation coefficients under probabilistic hesitant fuzzy environment and its application in clinical comprehensive evaluation of orphan drugs
Source: PLoS One. 2024 May 6;19(5):e0303042. doi: 10.1371/journal.pone.0303042 (PMC11073718; doi:10.1371/journal.pone.0303042)
Supplement: S10 Table — (DOC) [file pone.0303042.s010.doc]

**S10 Table. The decision matrix.**

|  | *C_1_* | *C_2_* | *C_3_* | *C_4_* |
| --- | --- | --- | --- | --- |
| *A_1_* | 0.89,1 | 0.60,0.75 | 0.75,0.88 | 0.78,1 |
| *A_2_* | 0.22,0.33,0.44 | 0.33,0.43,0.50,0.60 | 0.88 | 0.44,0.56,0.67 |
| *A_3_* | 0.67,0.78 | 0.33,0.43 | 0.63 | 0.11,0.22 |
| *A_4_* | 0.67,0.78 | 0.75,1.00 | 0.38,0.50 | 0.67,0.78 |
| *A_5_* | 0.78 | 0.43,0.50 | 0.75,0.88,1.00 | 0.44,0.56 |
