# Supplementary material for: A novel MCGDM technique based on correlation coefficients under probabilistic hesitant fuzzy environment and its application in clinical comprehensive evaluation of orphan drugs
Source: PLoS One. 2024 May 6;19(5):e0303042. doi: 10.1371/journal.pone.0303042 (PMC11073718; doi:10.1371/journal.pone.0303042)
Supplement: Attachment — Submitted filename: Response to Reviewers.doc [file pone.0303042.s011.doc]

**Original Manuscript ID:** PONE-D-23-33158

**Original Article Title: “A novel MCGDM technique based on correlation coefficients under probabilistic hesitant fuzzy environment and its application in clinical comprehensive evaluation of orphan drugs** ”

**To:** Plos One Editor

**Re:** Response to reviewers

Dear Editor,

Thank you for allowing a resubmission of our manuscript, with an opportunity to address the reviewers’ comments.

We are uploading (a) our point-by-point response to the comments (below) (Response to Reviewers), (b) an updated manuscript with yellow highlighting indicating changes (*Revised Manuscript with Track Changes*), and (c) a clean updated manuscript without highlights (*Manuscript).*

Best regards,

<Yubo Hu> et al.

**Reviewer#1, Concern # 1:** The language requires meticulous proofreading by a native speaker to meet the academic standards expected for publication.

**Author response:** We gratefully appreciate for your valuable suggestion.

**Author action:** According to the reviewer’s comment, We invited native English speaking editors to help us correct spelling and grammar mistakes in the article. Thank you again for your valuable comment on the paper.

**Reviewer#1, Concern # 2:** Moreover, the article lacks a distinctive presentation of novelty; there is a noted absence of a compelling argument that clearly defines what sets this research apart from existing studies, a delineation of its unique contributions, and a discussion of its limitations.

**Author response:** We gratefully appreciate for your valuable suggestion.

**Author action:** According to the reviewer’s comment, We divided the introduction and the literature review into two parts. In the introduction, we added a summary of the research gaps in existing studies. Then, in the literature review, we reviewed and discussed each research gap separately, aiming at the different research gaps summarized in the introduction. In addition, the existing research gaps are discussed, specifically in the first paragraph of the second part of the paper. Then, in terms of the research on the correlation coefficient of the existing probabilistic hesitancy fuzzy sets, we have discussed the shortcomings of the existing research in the paper before. Finally, in terms of the research on the multi-attribute decision making method, we added the literature review on the MCDM method. The existing MCDM methods are summarized and classified, and then the reasons for selecting the multi-attribute group decision making method based on correlation coefficient are discussed.

**Reviewer#1, Concern # 3:** The manuscript should offer a more expansive comparison to contemporary methods such as "PT-TOPSIS methods for multi-attribute group decision making under single-valued neutrosophic sets" or "EDAS method for multiple attribute group decision making under spherical fuzzy environment," highlighting both similarities and differences. In addition, a broader literature review and a more comprehensive background on MCDA would fortify the study's contextual relevance and scholarly depth**Author response:** We gratefully appreciate for your valuable suggestion.

**Author action:** According to the reviewer’s comment, We reviewed and classified the two literatures in the fourth paragraph of the second part

**Reviewer#1, Concern # 4:** The inclusion of a succinct comparison with methodologies such as SPOTIS, ESP-COMET, SIMUS, RANCOM, and others, is essential to demonstrate the robustness and relevance of the proposed MCGDM technique.

**Author response:** We gratefully appreciate for your valuable suggestion.

**Author action:** According to the reviewer’s comment, The main common advantage of the above mentioned methods is that they can overcome the phenomenon of rank reversal. In order to show that the ordering inversion phenomenon does not exist in our method, we add a test on the phenomenon of rank reversal in the seventh part to demonstrate the reliability of our proposed method.

**Reviewer#1, Concern # 5:** Furthermore, the research would benefit substantially from detailing the process of constructing the decision matrix and justifying the selection of the criteria involved.

**Author response:** We gratefully appreciate for your valuable suggestion.

**Author action:** According to the reviewer’s comment, We have added a description of attributes in Table 3 of Part VI. Second, the method decision process has been described in detail in previous articles.

**Reviewer#1, Concern # 6:** employing sensitivity analysis to assess the robustness of the proposed MCGDM method.

**Author response:** We gratefully appreciate for your valuable suggestion.

**Author action:** According to the reviewer’s comment, We added sensitivity analysis on attribute weights in 7.1 of Part 7 of the paper

**Reviewer#1, Concern # 7:** Additionally, expanding the number of alternatives considered in the case study would provide a more thorough examination of the method's applicability and reliability.

**Author response:** We gratefully appreciate for your valuable suggestion.

**Author action:** According to the reviewer’s comment, In the seventh part of this paper, 7.2, we test whether there is the phenomenon of rank reversal in our method by adding more alternatives, so as to show that our method will not affect the order of the previous schemes when adding alternatives, and thus demonstrate the reliability of our method.

**Reviewer#1, Concern # 8:** Finally, the introduction section demands elaboration to set the stage for the readers properly, providing them with a firm grasp of the research context, the prevailing challenges, and the envisioned solutions.

**Author response:** We gratefully appreciate for your valuable suggestion.

**Author action:** According to the reviewer’s comment, We divided the introduction and the literature review into two parts, in which we added a summary of the research gaps existing in the existing research and provided a brief summary of the work we had done.

**Reviewer#2, Concern # 1:** The authors do not provide sufficient motivation for the study

**Author response:** We gratefully appreciate for your valuable suggestion.

**Author action:** According to the reviewer’s comment, We divided the introduction and the literature review into two parts. In the introduction, we added a summary of the research gaps existing in the existing studies. Then in the literature review, we reviewed and discussed each research gap according to the different research gaps summarized in the introduction

**Reviewer#2, Concern # 2:** The authors do not benchmark their approach against reference approaches

**Author response:** We gratefully appreciate for your valuable suggestion.

**Author action:** According to the reviewer’s comment, in the seventh part, we added sensitivity analysis to our method and tested the ordering inversion phenomenon by referring to other decision-making methods that can effectively overcome the ordering inversion phenomenon. At last, we compared our method with the existing correlation coefficient and the MCDM method based on the correlation coefficient.

**Reviewer#3, Concern # 1:** 1. The literature review part is not just list literatures, you should find the research gap and the implications of your research through the literature review part. However, I can’t see it. I suggest author also should clarify the limitations of existing literatures more clearly, list as 1,2,3….Besides, I suggest adding a separate literature review section.

**Author response:** We gratefully appreciate for your valuable suggestion.

**Author action:** According to the reviewer’s comment, We divided the introduction and the literature review into two parts. In the introduction, we added a summary of the research gaps in existing studies. Then, in the literature review, we reviewed and discussed each research gap separately, aiming at the different research gaps summarized in the introduction. In addition, the existing research gaps are discussed, specifically in the first paragraph of the second part of the paper. Then, in terms of the research on the correlation coefficient of the existing probabilistic hesitancy fuzzy sets, we have discussed the shortcomings of the existing research in the paper before. Finally, in terms of the research on the multi-attribute decision making method, we added the literature review on the MCDM method. The existing MCDM methods are summarized and classified, and then the reasons for selecting the multi-attribute group decision making method based on correlation coefficient are discussed.

**Reviewer#3, Concern # 2:** 2. Although this article has been a comprehensive overview, Some classic methods should be mentioned, such as Best-Worst method (BWM), Weighted Aggregated Sum-Product Assessment (WASPAS), SMART (Simple Multi-attribute Rating Technique), DEMATEL (Decision-Making Trial and Evaluation Laboratory),etc. I suggest that the author needs to add relevant content to discuss the reasons why chose to use correlation coefficients this method.

**Author response:** We gratefully appreciate for your valuable suggestion.

**Author action:** According to the reviewer’s comment, We add a literature review of relevant MCDM methods, summarize and classify existing MCDM methods, and then discuss the reasons for our proposed multi-attribute group decision making method based on correlation coefficient

**Reviewer#3, Concern # 3:** 3. The comparison analysis between the proposed method and the existing method and the discussion of the results should be more in-depth.

**Author response:** We gratefully appreciate for your valuable suggestion.

**Author action:** According to the reviewer’s comment, In the seventh part, we added sensitivity analysis to our method and tested the ordering inversion phenomenon by referring to other decision-making methods that can effectively overcome the ordering inversion phenomenon. At last, we compared our method with the existing correlation coefficient and the MCDM method based on the correlation coefficient.

**Reviewer#3, Concern # 3:** 4. I noticed that some of the references were not convincing enough and suggested updating them.

**Author response:** We gratefully appreciate for your valuable suggestion.

**Author action:** According to the reviewer’s comment, In the second part, we have added some new literature to strengthen the argument of our article and updated some of the literature.

**Reviewer#3, Concern # 3:** 5. In the conclusion part, the limitations of this paper need to be discussed

**Author response:** We gratefully appreciate for your valuable suggestion.

**Author action:** According to the reviewer’s comment, In the conclusion, we add the limitations of our method and look forward to the future to address such limitations.
